# Integrating lymphovascular and perineural invasion into TNM staging: a novel ITNM system for enhanced prognostic stratification in colorectal cancer

**DOI:** 10.3389/fonc.2026.1745324

**Published:** 2026-01-28

**Authors:** Jiaqi Hu, Minghao Zhang, Yang Qi, Yun Wang, Dalin Xu, Kejin Zhu, Jun Bu

**Affiliations:** 1 Department of General Surgery, North Sichuan Medical College, School of Clinical Medicine, Nanchong, Sichuan, China; 2 Department of General Surgery, Chengdu Second People’s Hospital, Chengdu, Sichuan, China; 3 Department of General Surgery, Chengdu Medical College, Sichuan University Affiliated Chengdu Second People’s Hospital, Chengdu Second People’s Hospital, Chengdu, Sichuan, China; 4 Department of General Surgery, West China School of Medicine, Sichuan University, Sichuan University Affiliated Chengdu Second People’s Hospital, Chengdu Second People’s Hospital, Chengdu, Sichuan, China

**Keywords:** colorectal cancer, lymphovascular invasion, perineural invasion, prognosis model, TNM staging

## Abstract

**Background:**

The standard TNM staging system for colorectal cancer (CRC) fails to reflect true tumor biology because it ignores key histopathologic features such as lymphovascular and perineural invasion. We therefore developed and validated an “ITNM” classification that folds these two factors into the N stage, yielding more accurate prognoses and greater clinical value.

**Methods:**

A retrospective cohort of 442 stage I–III CRC patients underwent radical resection (2015–2021). Propensity score matching (PSM) created balanced exposure (LVI/PNI-positive, n = 185) and control (LVI/PNI-negative, n = 257) groups. The ITNM system was constructed by upstaging the N category based on LVI/PNI status. Predictive performance was evaluated using C-index, ROC-AUC, calibration curves, decision curve analysis (DCA), net reclassification improvement (NRI), and integrated discrimination improvement (IDI).

**Results:**

Multivariate analysis confirmed LVI (HR = 2.10, p = 0.018) and PNI (HR = 2.28, p = 0.025) as independent predictors of overall survival (OS). The LVI/PNI-positive group had significantly lower 5-year OS (69.5% *vs*. 88.2%, p < 0.001). The ITNM system demonstrated superior discriminative ability for 5-year OS (C-index = 0.715; AUC = 0.735, 95% CI: 0.691–0.776), excellent calibration (p = 0.489), and higher net benefit on DCA. Significant reclassification improvement was confirmed (NRI = 0.306, *p* < 0.001; IDI = 0.061, *p* < 0.001).

**Conclusion:**

The ITNM system significantly enhances prognostic accuracy by integrating LVI and PNI into TNM staging, enabling risk-adapted therapeutic decision-making and representing a paradigm shift in CRC stratification.

## 1 Introduction

CRC is one of the most common and lethal malignancies worldwide. In the United States, it is projected to rank third in both incidence and mortality among all major cancer types by 2024, according to the American Cancer Society (1). In China, CRC is similarly a significant public health concern, with an estimated 517,100 new cases in 2022, accounting for 10.7% of all newly diagnosed malignancies. It ranks second in incidence and fourth in mortality among all cancers, making it a leading cause of cancer-related death in the country (2). The Tumor Node Metastasis (TNM) system is the globally accepted method for staging CRC, guiding postoperative treatment plans. However, patients with the same TNM stage can have differing prognoses. In China, the average five-year survival rate for CRC patients is 56.9% (3), yet 30% to 50% of patients experience local recurrence or metastasis after surgery (4). The five-year survival rate drops significantly to 10.6% among those with metastatic disease (5). This variation in patient outcomes highlights the need for more precise prognostic indicators (6).

LVI and PNI are common pathological features observed in patients with CRC. LVI refers to the spread of tumor cells along the walls of blood vessels or lymphatic vessels (7), while PNI indicates the invasion of nerve sheaths or fibers by tumor cells. Both LVI and PNI are associated with increased tumor aggressiveness, recurrence, and poor prognosis. They have been identified as independent risk factors for adverse outcomes in CRC patients (8). Despite their clinical significance, LVI and PNI are currently not incorporated into the TNM staging system, and there is no consensus on their role in guiding patient management.

This study proposes a new clinical staging system, the ITNM (Invasion, Tumor, Node, Metastasis), which integrates LVI and PNI into the existing TNM framework. By assessing the predictive value of this revised staging system, this research aims to enhance the TNM system’s ability to inform treatment decisions, providing a more comprehensive approach to the management of CRC patients.

## 2 Materials and methods

### 2.1 Study population and design

**Figure 1:**
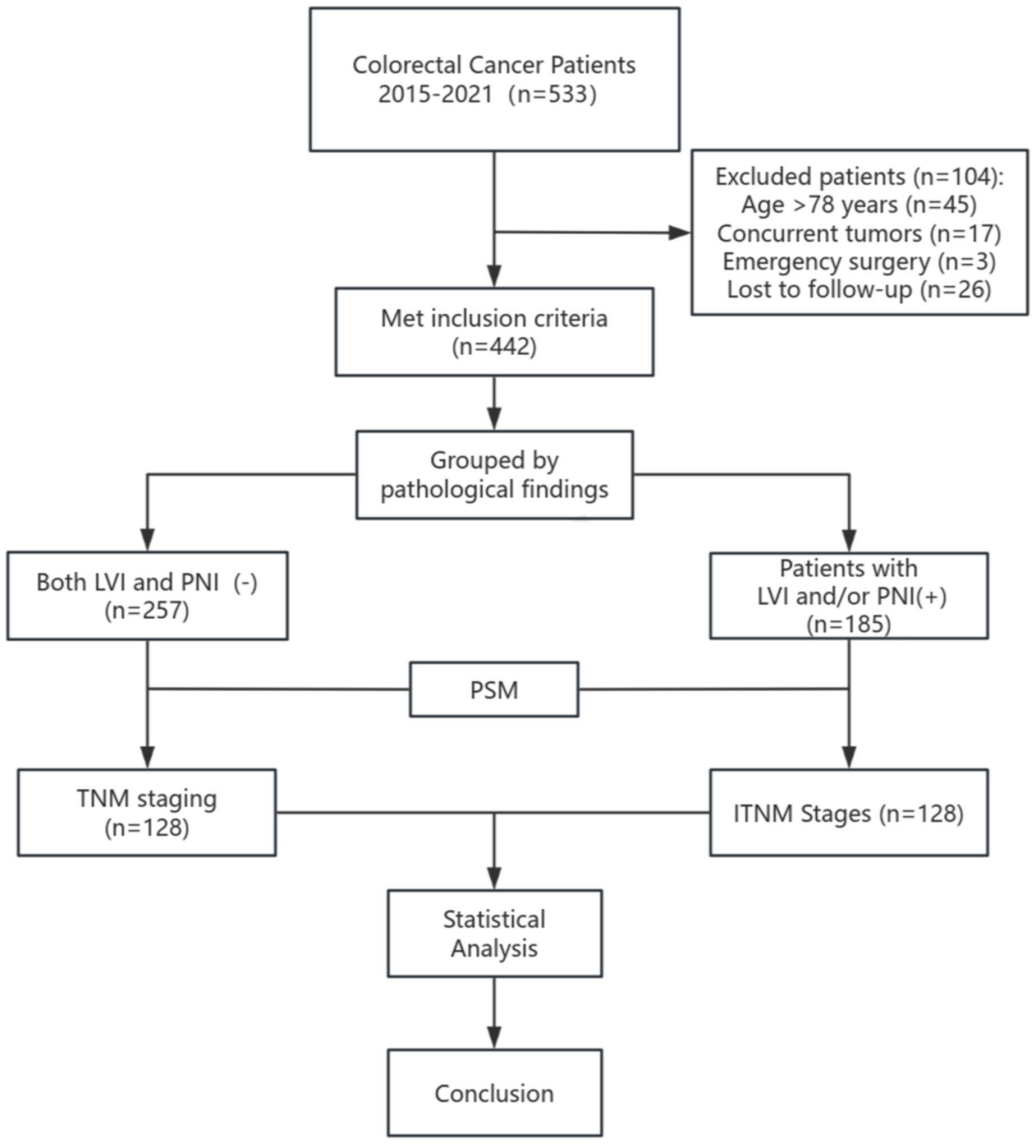
Flowchart of colorectal cancer patients.

This study was approved by the Ethics Committee of Chengdu Second People’s Hospital (Ethics Approval Number: [KY]PJ2024224). Given its retrospective design, informed consent was not required. The study consecutively included patients with colorectal cancer who underwent radical surgery at Chengdu Second People’s Hospital between January 2015 and December 2021. Demographic data, pathological findings, and disease status at the latest follow-up were obtained from hospital records. Inclusion criteria were as follows: (1) post-operative pathology confirming colorectal adenocarcinoma, (2) complete and available patient records, (3) age between 18 and 78 years, (4) preoperative signed surgical and informed consent forms, (5) no prior neoadjuvant or other anticancer treatments before surgery, and (6) cTNM stage I-III (American Joint Committee on Cancer/Union for International Cancer Control 8th Edition). Exclusion criteria included: (1) severe cardiac, pulmonary, cerebral, or other major functional impairments, (2) palliative tumor resection, (3) history of other concurrent malignancies, (4) emergency surgery, and (5) uncontrolled psychiatric conditions or patients lacking full legal capacity. Patients were classified into two groups: the control group (no LVI or PNI based on pathology) and the exposure group (LVI/PNI present). To minimize the influence of confounding factors in this retrospective study, propensity score matching was used (**Figure 1**).

### 2.2 Pathological assessment and variable definitions

According to the AJCC/UICC TNM staging system, two or more experienced pathologists re-examine resected primary tumor specimens. Through hematoxylin and eosin (HE) staining, they determine the tumor location within the specimen, tumor diameter, histological type, degree of differentiation, number of lymph nodes cleared, and the presence of vascular and nerve invasion. Vascular invasion is defined as the presence of tumor cells within the vascular muscular layer or the invasion of muscular vascular endothelium. Lymphatic invasion is defined as the presence of tumor cell clusters within non-muscular lymphatic lumens (9). PNI is defined as the presence of tumor cells within all three layers of the nerve sheath, or adjacent to the nerve, affecting at least 33% of its entire circumference (10).

To stratify continuous variables, we referred to clinical guidelines and prior experience, considering factors such as patient survival rates, treatment tolerance, and the risks associated with increased tumor malignancy and metastasis. The following cutoff points were applied for stratification: age ≥65 years, tumor diameter ≥5 cm (11), lymph node dissection of at least 12 nodes (12). Anatomically, the right colon includes the cecum, ascending colon, hepatic flexure, and transverse colon, while the left colon comprises the splenic flexure, descending colon, and sigmoid colon.

### 2.3 Construction of the ITNM staging system

The incorporation of LVI and PNI into the N staging involves a modified weighting system. Specifically, the presence of either LVI or PNI results in an increase of one stage in the N category. This adjustment leads to the development of new N categories, which, in turn, are integrated into an updated TNM staging system. The revised system is referred to as the ITNM (Invasion-Tumor-Node-Metastasis) staging system, with the new IN category outlined in **Table 1**.

**Table 1:** ITNM staging system.

	LVI(+)	PNI(+)	LVI(+)PNI(+)
Installment			
N0	IN1	IN1	IN2
N1	IN2	IN2	IN3
N2	IN3	IN3	IN3

### 2.4 Statistical analysis

Data was collected using Excel, analyzed with SPSS 29.0, and visualized using R 4.4.2. Qualitative data are presented as frequencies (n%) and were analyzed using chi-square or Fisher’s exact tests, as appropriate. The Log-rank test was used for significance analysis, while survival data were analyzed using the Kaplan-Meier method. Independent risk factors influencing prognosis were identified through univariate and multivariate Cox regression models. The performance of the new model was assessed using the C-index, receiver operating characteristic (ROC) curve, calibration curve, and DCA. The NRI and IDI were used to quantify the improvement of the new ITNM prediction model over the baseline model. A p-value of < 0.05 was considered statistically significant.

## 3 Results

### 3.1 Patient characteristics

A total of 442 patients were included in the study, with a mean age of 66 years. Among them, 257 (58.1%) were male, and 185 (41.8%) were female. The median follow-up period was 60 months. During this time, 80 patients (18%) died, and 111 (25.1%) experienced recurrence. The rate of postoperative chemotherapy was 40.9%. In terms of tumor pathology, 41.1% of patients were diagnosed with stage I, 36.8% with stage II, and 21.9% with stage III disease. Most tumors were moderately differentiated (58.1%), and 59.5% of the tumors were less than 5 cm in diameter. Additionally, 19.6% of patients had concurrent tumor deposits (TD). Patients were divided into two groups based on the presence or absence of LVI or PNI in their pathological findings. The control group consisted of 257 patients (58.1%), and the exposure group included 185 patients (41.8%). A total of 49 patients were classified in the IN3 stage, representing 11.0% of the cohort. Among them, 24 patients (51.0%) died, and 27 (55.1%) experienced disease relapse. Significant differences were observed between the two groups in pTNM stage, degree of differentiation, tumor deposits (TD), and postoperative chemotherapy (p <.001). After propensity score matching (PSM), which yielded 128 patients in each group, the baseline clinical and pathological characteristics were well-balanced, with no significant differences observed (**Table 2**).

**Table 2:** Baseline characteristics of colorectal cancer patients.

Variables	Subgroup	Before PSM			After PSM		
		Control group (n = 257)	Exposure group (n = 185)	*P*	Control group (n = 128)	Exposure group (n = 128)	*P*
Age, n (%)	<65	118 (45.91)	79 (42.70)	0.503	57 (44.53)	56 (43.75)	0.900
	≥65	139 (54.09)	106 (57.30)		71 (55.47)	72 (56.25)	
Gender, n (%)	Male	155 (60.31)	110 (59.46)	0.857	77 (60.16)	75 (58.59)	0.799
	Female	102 (39.69)	75 (40.54)		51 (39.84)	53 (41.41)	
BMI, n (%)	≤28	240 (93.39)	172 (92.97)	0.865	118 (92.19)	122 (95.31)	0.302
	>28	17 (6.61)	13 (7.03)		10 (7.81)	6 (4.69)	
Smoking, n (%)	No	189 (73.54)	128 (69.19)	0.316	92 (71.88)	87 (67.97)	0.496
	Yes	68 (26.46)	57 (30.81)		36 (28.12)	41 (32.03)	
Alcohol Consumption, n (%)	No	215 (83.66)	143 (77.30)	0.093	103 (80.47)	100 (78.12)	0.644
	Yes	42 (16.34)	42 (22.70)		25 (19.53)	28 (21.88)	
Preoperative Complications, n (%)	None	142 (55.25)	99 (53.51)	0.717	68 (53.12)	68 (53.12)	1.000
	Present	115 (44.75)	86 (46.49)		60 (46.88)	60 (46.88)	
Tumor Location, n (%)	Left-sided	48 (18.68)	37 (20.00)	0.886	26 (20.31)	25 (19.53)	0.536
	Right-sided	60 (23.35)	40 (21.62)		35 (27.34)	28 (21.88)	
	Rectum	149 (57.98)	108 (58.38)		67 (52.34)	75 (58.59)	
pTNM Stage, n (%)	I	80 (31.13)	17 (9.19)	**<.001**	13 (10.16)	17 (13.28)	0.485
	II	126 (49.03)	56 (30.27)		64 (50.00)	55 (42.97)	
	III	51 (19.84)	112 (60.54)		51 (39.84)	56 (43.75)	
Tumor Diameter, n (%)	<5cm	159 (61.87)	104 (56.22)	0.232	72 (56.25)	73 (57.03)	0.900
	≥5cm	98 (38.13)	81 (43.78)		56 (43.75)	55 (42.97)	
Tumor Type, n (%)	Ulcerative	140 (54.47)	117 (63.24)	0.168	81 (63.28)	76 (59.38)	0.802
	Elevated	115 (44.75)	67 (36.22)		46 (35.94)	51 (39.84)	
	Infiltrative	2 (0.78)	1 (0.54)		1 (0.78)	1 (0.78)	
Degree of Differentiation, n (%)	High	55 (21.40)	19 (10.27)	**<.001**	13 (10.16)	12 (9.38)	0.910
	Moderate	156 (60.70)	101 (54.59)		80 (62.50)	78 (60.94)	
	Low	46 (17.90)	65 (35.14)		35 (27.34)	38 (29.69)	
Total dissected lymph nodes, n (%)	<12	156 (60.70)	95 (51.35)	0.050	66 (51.56)	74 (57.81)	0.315
	≥12	101 (39.30)	90 (48.65)		62 (48.44)	54 (42.19)	
TD, n (%)	Absent	230 (89.49)	125 (67.57)	**<.001**	102 (79.69)	95 (74.22)	0.299
	Present	27 (10.51)	60 (32.43)		26 (20.31)	33 (25.78)	
Postoperative Chemotherapy, n (%)	No	173 (67.32)	88 (47.57)	**<.001**	70 (54.69)	68 (53.12)	0.802
	Yes	84 (32.68)	97 (52.43)		58 (45.31)	60 (46.88)	

Bold values indicate that the difference is statistically significant (*P* < 0.01).

### 3.2 Prognostic impact of LVI and PNI

Univariate Cox regression analysis identified several factors associated with poorer 5-year overall survival in colorectal cancer patients, including TD (*p* < 0.001), LVI (*p* = 0.003), and PNI (*p* < 0.001). In the multivariate analysis, TD (*p* < 0.001), LVI (*p* = 0.018), and PNI (*P* = 0.025) were identified as independent risk factors for OS (**Table 3**).

**Table 3:** Cox univariate and multivariate regression analysis for 5-year OS.

Variables	Subgroup	Univariate		Multivariate	
		HR (95%CI)	*P*	HR (95%CI)	*P*
TD	Absent	1.00 (Reference)		1.00 (Reference)	
	Present	3.05 (1.78 ~ 5.21)	**<.001**	2.63 (1.50 ~ 4.58)	**<.001**
LVI	Absent	1.00 (Reference)		1.00 (Reference)	
	Present	2.28 (1.32 ~ 3.94)	**0.003**	1.98 (1.13 ~ 3.49)	**0.018**
PNI	Absent	1.00 (Reference)		1.00 (Reference)	
	Present	2.86 (1.66 ~ 4.93)	**<.001**	1.94 (1.09 ~ 3.47)	**0.025**

Bold values indicate that the difference is statistically significant (*P* < 0.01).

For the 5-year DFS, univariate analysis revealed significant predictors, including Total dissected lymph nodes (*p* = 0.027), pTNM Stage (*p* = 0.005), TD (*p* < 0.001), LVI (*p* = 0.003), and PNI (*p* < 0.001) were identified as independent risk factors for DFS. After adjusting for these factors in a multivariate Cox proportional hazards model, pTNM Stage (*p* = 0.041), TD (*p* = 0.006), LVI (*p* = 0.020), and PNI (*p* = 0.043) were found to be independent risk factors for 5-year DFS (**Table 4**).

**Table 4:** Cox univariate and multivariate regression analysis for 5-year DFS.

Variables	Subgroup	Univariate		Multivariate	
		HR (95%CI)	*P*	HR (95%CI)	*P*
Total dissected lymph nodes	<12	1.00 (Reference)		1.00 (Reference)	
	≥12	0.59 (0.36 ~ 0.94)	**0.027**	0.82 (0.49 ~ 1.37)	0.449
pTNM Stage	I	1.00 (Reference)		1.00 (Reference)	
	II	2.49 (0.76 ~ 8.20)	0.133	2.83 (0.85 ~ 9.41)	0.089
	III	5.33 (1.66 ~ 17.12)	**0.005**	3.57 (1.05 ~ 12.09)	**0.041**
TD	Absent	1.00 (Reference)		1.00 (Reference)	
	Present	3.44 (2.20 ~ 5.38)	**<.001**	2.21 (1.25 ~ 3.89)	**0.006**
LVI	Absent	1.00 (Reference)		1.00 (Reference)	
	Present	1.95 (1.25 ~ 3.06)	**0.003**	1.74 (1.09 ~ 2.78)	**0.020**
PNI	Absent	1.00 (Reference)		1.00 (Reference)	
	Present	2.70 (1.70 ~ 4.27)	**<.001**	1.68 (1.02 ~ 2.78)	**0.043**

Bold values indicate that the difference is statistically significant (*P* < 0.01).

### 3.3 Relationship between LVI and PNI with OS and DFS

**Figure 2:**
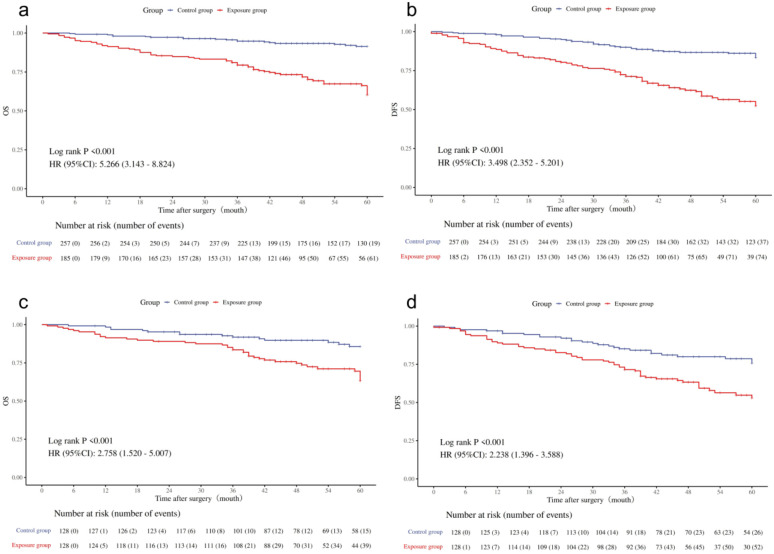
Survival curves for the exposed group and the control group. **(a)** Overall survival curve before PSM. **(b)** Disease-free survival curve before PSM. **(c)** Overall survival curve after PSM. **(d)** Disease-free survival curve after PSM.

Kaplan-Meier survival curves, both before and after propensity score matching (PSM), illustrate the impact of LVI and PNI on the prognosis of colorectal cancer patients. In the unadjusted analysis, the 5-year OS and DFS rates were both lower in patients with LVI/PNI compared to those without these invasions (**Figures 2a, b**).

After propensity score matching, the 5-year OS for patients with LVI/PNI was 69.5%, significantly lower than the 88.2% observed in the control group (*p* < 0.001. Similarly, the 5-year DFS in the exposed group was 59.3%, compared to 79.6% in the control group (*p* < 0.001) (**Figures 2c, d**). These results highlight that patients with positive LVI/PNI have a poorer prognosis than those without these invasions.

### 3.4 Predictive performance of the ITNM staging system

**Figure 3:**
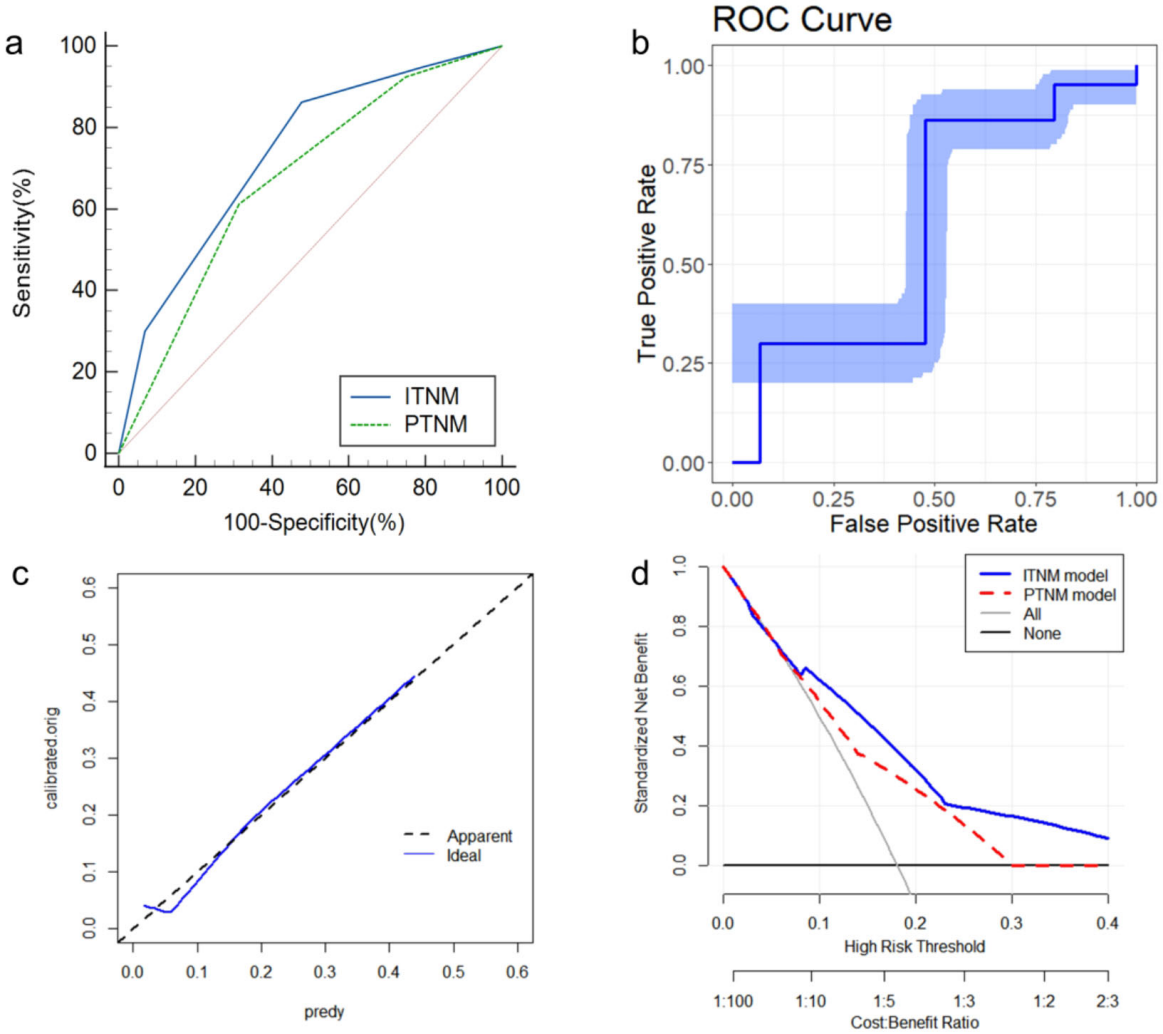
Comparison and Validation of the ITNM Staging System. **(a)** ROC curve of the ITNM model. **(b)** Bootstrap ROC curve of the ITNM model. **(c)** Calibration curve of the ITNM model. **(d)** Decision-curve analysis of the ITNM model.

The proposed ITNM system demonstrated excellent predictive performance. The C-index for predicting 5-year OS was 0.715. The area under the ROC curve (AUC) was 0.735 (95% CI: 0.691-0.776), which was significantly higher than that of the pTNM system (*p* = 0.004) (**Figure 3a**). Bootstrap validation with 1000 resamples confirmed the robustness of this AUC estimate (0.735, 95% CI: 0.676-0.787) (**Figure 3b**). The calibration curve showed strong agreement between predicted and observed survival probabilities (**Figure 3c**), which was confirmed by a non-significant Hosmer-Lemeshow test (*p* = 0.489). Most importantly, DCA revealed that the ITNM system provided a superior net benefit compared to the “treat-all” or “treat-none” strategies across a wide range of threshold probabilities, underscoring its clinical utility (**Figure 3d**).

### 3.5 Net reclassification improvement analysis

The key advancement of the ITNM system is quantified by the NRI analysis. Compared to the traditional pTNM staging, the ITNM system resulted in a significant categorical NRI of 0.306 (95% CI: 0.188-0.423, *p* < 0.001), indicating that 30.6% of patients were correctly reclassified into more accurate risk categories. The continuous NRI was 0.611 (95% CI: 0.376-0.846, *p* < 0.001). Furthermore, the IDI was 0.061 (95% CI: 0.035-0.088, *p* < 0.001), demonstrating a significant improvement in the model’s overall discriminatory power. These results robustly confirm that the ITNM system offers a substantive improvement in risk stratification accuracy.

## 4 Discussion

This study developed and validated an ITNM staging system that incorporates LVI and PNI. The findings reveal that patients with positive LVI/PNI markers have a notably worse prognosis, with both factors identified as independent predictors of OS in CRC. The new ITNM system outperforms the traditional TNM staging in its ability to distinguish between patient outcomes and offers greater clinical benefit in predicting OS. These results underscore the system’s potential value in evaluating postoperative survival and guiding decisions regarding the need for adjuvant therapy in CRC patients.

The American Joint Committee on Cancer (AJCC) TNM staging system is widely used in clinical practice to assess colorectal cancer prognosis. This system primarily evaluates three key factors: the depth of the primary tumor invasion (T), the presence of lymph node metastasis (N), and the presence of distant metastasis (M) (13). However, as clinical research progresses, the limitations of the traditional TNM system have become evident. One major drawback is its tendency to categorize patients within the same stage as homogenous, despite significant variations in their risk profiles. For example, all stage II colon cancer patients without lymph node metastasis are grouped, yet their recurrence risks may differ substantially. Relying solely on T and N status often fails to identify high-risk patients within this group (14, 15), as illustrated by a T3 patient with no lymph node metastasis (N0) but with LVI or PNI, who may have a higher risk than some N1 patients without LVI or PNI (16). Traditional TNM staging also overlooks important histopathological factors such as LVI and PNI, both of which are established independent prognostic indicators in colorectal cancer (17). Numerous studies consistently show that the presence of either LVI or PNI, irrespective of cancer stage (18), is associated with significantly poorer OS and DFS (19, 20) 18. As a result, there has been growing interest in integrating these factors into the TNM staging system to improve risk stratification. Our study aligns with this effort, although the specific strategies vary. Similar to Yamano et al.’s approach (21), which incorporates LVI alongside tumor markers for risk stratification, we confirmed the independent prognostic value of LVI. However, our study goes further by including both LVI and PNI as weighted factors within the N stage, creating a more structured ITNM system. This approach mirrors Yang et al.’s model (22), which integrates multiple pathological features, including PNI, to improve the predictive accuracy of the staging system. Further supporting this approach, studies by Kang et al (23–25) demonstrated that combining LVI, PNI, and other histological risk factors provides a more accurate prognosis. However, while these studies grouped these factors independently, our methodology innovatively incorporates them into the N stage to enhance risk stratification. This aligns with the “TVI” (T staging + venous invasion) staging system proposed by Roxburgh et al (26), which aims to include additional critical prognostic information, thereby improving the staging system’s overall predictive value.

In the context of ITNM staging, the presence of LVI or PNI in the pathology report indicates the need for combined chemotherapy regimens, contrasting with the current stratified approach for high-risk stage II colorectal cancer. The influence of LVI and PNI on guiding adjuvant therapy has been well-documented. A multicenter study by Li et al (27), involving 931 stage II colon cancer patients, highlighted a significant interaction between PNI status and adjuvant chemotherapy (ACT), showing that PNI-positive patients experience a notably greater survival benefit from ACT compared to those who are PNI-negative. Similarly, a large-scale study by Song et al (28) found that while both LVI and PNI are associated with a poor prognosis, PNI plays a more substantial role in guiding postoperative adjuvant therapy. These findings suggest that PNI status may be a more critical determinant in deciding whether additional systemic adjuvant chemotherapy is necessary. Despite ongoing debate about the role of adjuvant therapy for LVI/PNI-positive patients, an increasing number of studies and clinical guidelines now support the consideration of LVI and PNI positivity as significant high-risk factors in the decision-making process for adjuvant chemotherapy in colorectal cancer (29).

This study is, to our knowledge, the first to explore the prognostic significance of a novel histological grading system that integrates LVI, PNI, and N staging in colorectal cancer. This risk stratification approach could improve the individualized management of patients undergoing colorectal cancer surgery. Despite advancements in research, formally incorporating LVI and PNI into the TNM staging system continues to pose challenges, primarily due to the discrepancies in definitions, which necessitate a global consensus among pathologists for cohesive reporting. Furthermore, the limitations of this study — being single-centered with a lengthy retrospective design and potentially confounding variables such as evolving surgical techniques and tumor markers — underscore the necessity for larger, multicenter prospective trials to validate the prognostic value of the proposed new staging system. Additionally, the relatively small sample size restricts the generalizability of our findings, highlighting the urgent need for further research into the interplay between LVI/PNI, molecular markers, and microscopic pathological features. Such investigations aim to define patient subgroups that would benefit most from this advanced staging system, ultimately propelling the field toward the goals of personalized, precision medicine.

In considering the range of prognostic factors that inform precision medicine, Mismatch Repair (MMR) status emerges as a cornerstone biomarker in the diagnosis and treatment of colorectal cancer. The 2017 guidelines from the Chinese Society of Clinical Oncology (CSCO) advocate for MMR/MSI testing in all newly diagnosed colorectal cancer patients, emphasizing its role in guiding Lynch syndrome screening and assessing prognosis. The multifaceted importance of MMR status — encompassing prognosis prediction, treatment guidance, genetic screening, and immunotherapy — highlights a critical dimension of patient management that spans the trajectory from initial diagnosis to advanced therapeutic interventions. Although this study did not evaluate MMR status, its potential role warrants exploration as a crucial component of future iterations of the ITNM system. Integrating MMR status alongside LVI and PNI can provide a more nuanced understanding of tumor biology and patient outcomes, further enriching the objectives of personalized, precision medicine.

## 5 Conclusion

In conclusion, LVI and PNI are important independent prognostic factors for colorectal cancer. When combined with N staging, these factors enhance the accuracy of risk assessment, overcoming the limitations of the current TNM staging system. This integrated approach allows for more tailored treatment strategies, ultimately leading to improved patient outcomes.

## Data Availability

The original contributions presented in the study are included in the article/supplementary material. Further inquiries can be directed to the corresponding author.
